# Persistence and Dissipation of Chlorpyrifos in Brassica Chinensis, Lettuce, Celery, Asparagus Lettuce, Eggplant, and Pepper in a Greenhouse

**DOI:** 10.1371/journal.pone.0100556

**Published:** 2014-06-26

**Authors:** Meng-Xiao Lu, Wayne W. Jiang, Jia-Lei Wang, Qiu Jian, Yan Shen, Xian-Jin Liu, Xiang-Yang Yu

**Affiliations:** 1 Pesticide Biology and Ecology Research Center, Nanjing, Jiangsu, China; 2 Key Laboratory of Food Safety Monitoring and Management of Ministry of Agriculture, Nanjing, Jiangsu, China; 3 Department of Entomology, Michigan State University, East Lansing, Michigan, United States of America; 4 Institute for the Control of Agrochemicals, Ministry of Agriculture, Beijing, China; Institute of Vegetables and Flowers, Chinese Academy of Agricultural Science, China

## Abstract

The residue behavior of chlorpyrifos, which is one of the extensively used insecticides all around the world, in six vegetable crops was assessed under greenhouse conditions. Each of the vegetables was subjected to a foliar treatment with chlorpyrifos. Two analytical methods were developed using gas chromatography equipped with a micro-ECD detector (LOQ = 0.05 mg kg^−1^) and liquid chromatography with a tandem mass spectrometry (LOQ = 0.01 mg kg^−1^). The initial foliar deposited concentration of chlorpyrifos (mg kg^−1^) on the six vegetables followed the increasing order of brassica chinensis<lettuce<celery<asparagus lettuce<eggplant <pepper. The initial deposition of chlorpyrifos showed differences among the six selected vegetable plants, ranging from 16.5±0.9 mg kg^−1^ (brassica chinensis) to 74.0±5.9 mg kg^−1^ (pepper plant). At pre-harvest interval 21 days, the chlorpyrifos residues in edible parts of the crops were <0.01 (eggplant fruit), <0.01 (pepper fruit), 0.56 (lettuce), 0.97 (brassica chinensis), 1.47 (asparagus lettuce), and 3.50 mg kg^−1^ (celery), respectively. The half-lives of chlorpyrifos were found to be 7.79 (soil), 2.64 (pepper plants), 3.90 (asparagus lettuce), 3.92 (lettuce), 5.81 (brassica chinensis), 3.00 (eggplant plant), and 5.45 days (celery), respectively. The dissipation of chlorpyrifos in soil and the six selected plants was different, indicating that the persistence of chlorpyrifos residues strongly depends upon leaf characteristics of the selected vegetables.

## Introduction

Chlorpyrifos [*O*,*O*-diethyl *O*-(3,5,6-trichloro-2-pyridyl) phosphorothioate] is an organophosphorous insecticide, acaricide, and nematicide used to control a broad spectrum of foliage and soil-born insect pests on a variety of food and feed crops [1]–[2]. It is ranked as one of the most extensively used insecticides all around the world. In China, since the use of several highly-toxic organophosphorous insecticides was banned in 2006, chlorpyrifos has been recommended as one of the alternative insecticides and broadly used in agriculture. Extensive use of chlorpyrifos has led to a potential risk of residues in various crops. Chlorpyrifos is of great environmental concerns due to its widespread use in the past several decades and its potential toxic effects on human health. Thus, the degradation study of chlorpyrifos has become increasing important in recent years [3]–[5]. In a market monitoring study conducted between 2007 and 2010, chlorpyrifos was detected in approximately 22.8% of 2082 samples of 17 vegetable commodities collected from Zhejiang Province, China with a highest residue of 3.47 mg kg-1. The residue levels in 1.4% of vegetable samples were found to be higher than the maximum residue limits (MRLs) of China [3].

Although most pesticides are effective to control pests in agricultural industry, inappropriate uses of pesticides may lead to public concerns on food safety and human health [5]–[10], environmental contamination [11]–[12], insect resistance and resurgence [12]–[14], etc. In China, the use of agrochemicals is critical to provide food supplies for its growing population with its limited arable land. Ideally, control the harmful organism efficiently, having no or minimum pesticide residues in the harvested crops, or at least lower than the statutory MRLs [3], [15]. Therefore, the field dissipation studies of pesticide persistence in foods and pesticide residue behavior is of particular importance in order to find out which pesticide application strategies are efficient to control insect pests while leaving minimum residues [16], [17]. There are many factors that influence the dissipation behavior of pesticides in plants, including the climate conditions (temperature, humidity, light intensity, etc) [18], the crop species [19]–[22] the nature of the chemicals, the formulations, and the application methods [23]–[24]. Due to the difference in the extension of the foliar, which resulted in the different initial pesticide deposition, and/or the difference in the metabolism system of different crops, dissipation of a pesticide on various crops may be markedly different [25]–[26]. A dissipation study of pesticides on leafy vegetables showed that among the tested vegetables, spinach and amaranth could incur higher pesticide deposition and that half-lives (*t*_1/2_ = 1.37–5.17 days) of chlorpyrifos were from on different leafy vegetables [22]. For chiral pesticide malathion, the calculated *t*_1/2_ values of the enantiomers were relatively short, ranging from 0.83 to 1.43 days in five plants. The degradation of the two enantiomers in Chinese cabbage (brassica chinesis), rape, and sugar beet was highly selective, while non-enantioselectivity was found in paddy rice and wheat [20]–[21]. For better understanding the possible residue risk of a pesticide, dissipation studies for different crop species in the specific growing conditions are necessary to test if the established application strategies are suitable.

It is concluded that agrochemicals enter into plants via two major pathways, which are either via foliar treatment - foliar deposition followed by entering into the inner parts of the crops, or via soil treatment - root uptake from the soil [27]–[28]. For the foliar pesticide applications, the agrochemicals are deposited directly on foliar surfaces of the crops and the excess agrochemicals will precipitate in the soil. The resulting deposition fractions are determined predominantly by crop species, growth stage of crop, pesticide formulation, and spraying technology [29]. Generally, optimization of chlorpyrifos uses is foliar application with minimizing losses of the applied pesticides from plants to soil. Spray loss of chlorpyrifos may lead to the soil environment pollution and extend its residue duration in plants. The chlorpyrifos residues were found to persist in soils, as the half-lives ranged generally between 50 and 120 days [30]–[32]. It was reported in the literature that the chlorpyrifos residues were found in soils for over one year after the applications. Pesticide persistence in soils may depend on the formulation, rate of application, soil type, climate, and other conditions [33]–[37]. Plant rhizosphere plays an important role in the degradation of pesticides in soils [21], [36].

In the present study, six vegetables were selected, including two fruit vegetables (pepper and eggplant) and 4 whole plant edibles leafy vegetables (brassica chinensis, lettuce, celery, and asparagus lettuce). They were treated with chlorpyrifos by foliar application under the controlled conditions in a greenhouse. The initial deposition of chlorpyrifos on the crop foliar was analyzed by GC and on the fruits by LC/MS/MS and the dynamics of pesticide residues in the plants and rhizosphere soils were to be monitored. The work was to evaluate the persistence and dissipation behavior of chlorpyrifos in different vegetables and soil. The results would help to provide understanding of the residue characteristics of chlorpyrifos in vegetables, and help to guide proper and safe use of pesticides on vegetables to ensure food safety.

## Materials and Methods

### Instruments and Reagents

Gas chromatography was an Agilent GC 7890 (Agilent Technologies, Santa Clara, CA, USA) equipped with a micro-ECD detector and an analytical column HP-5ms J&W Ultra Inert capillary column (30 m length×0.25 mm I.D.×0.25 µm film thickness, Agilent Technologies, USA). LC/MS/MS (Agilent Technologies, USA) contained a 1200 SL HPLC system coupled to an Agilent G6410A triple quadrupole mass spectrometer. The column was an Agilent ZORBAX SB-C18 (2.1×150 mm, 5 µm) analytical column. Chlorpyrifos-EC 40% (Hubei Xian Long Chemical Co., LTD) was purchased from a local pesticide store. The analytical reference substance, chlorpyrifos (certified analytical standard, 99.7%) was purchased from National Standard Company (Tianjin, China). Two stock solutions (1000 mg L^−1^) were prepared by dissolving the chlorpyrifos standard (100 mg) in 100 mL of acetone (for GC) and acetonitrile (for LC/MS/MS), respectively. Working solutions were prepared by diluting the stock solution or a working solution using the organic solvents (acetone for GC and acetonitrile for LC/MS/MS). Acetone, n-hexane, acetonitrile, sodium chloride, and anhydrous sodium sulfate were of analytical grades, purchased from Kermel Chemical Reagent Co., Ltd (Tianjin, China). The 0.22 µm SCAA-104 membranes and 500 mg florisil SPE cartridges were purchased from Anpel Scientific Instrument Co., Ltd (Shanghai, China).

The six selected vegetables were brassica chinensis (*Brassicachinensis L.*), lettuce (*Lactuca sativa* spp), pepper (*Capsicum annuum* spp), eggplant (*Solanum melongena* L), celery (*Apium graveolens*), and asparagus lettuce (*Asparagus Lettuce* spp). The pepper and eggplant seeds were grown in a nursery tray before being transplanted to the field at the 2–3 leaf stage seedlings, and the other four vegetables were directly sowed in the field. The row spacing and inter-plant spacing were set to be 30 cm for the vegetables, except for celery which was sowed in rows with row spacing of 30 cm.

### Experiment design

Field experiments were conducted in a controlled environment in a greenhouse at the Experiment Station of Jiangsu Academy of Agricultural Science (JAAS), Nanjing, China from September to November 2013. JAAS permitted the study in its greenhouse and this study did not use protected area of land or sea, neither with relevant protected wildlife. Since the work was completed in the greenhouse, no specific permission was required. This study did not involve endangered or protected species. The soil was of sandy loam texture dried and its contents contained 30% of sand, 53% silt, 15% clay, and 2% organic matter. There were four 30 m^2^ trial plots to be selected for each vegetable, i.e., three replicates and one control. Between the plots there was a buffer strip of 0.5 m in width. Chlorpyrifos-EC 40% was applied by foliar spraying at a rate of 0.97 kg a.i./ha on October 21, 2013. At the time of spraying, brassica chinensis, lettuce, and asparagus lettuce were at the stage when the leaves overspread; celery was 15–16 cm of height and the leaves scattered; pepper and eggplant were at the stages of 30–40 cm of height and the leaves of the adjacent plants touched each other and overlapped. In this study, the plants were allowed to the leaves to overlap to cover most of the surface of soil and thus the loss of pesticides sprayed was minimum. The temperature inside the greenhouse was controlled between 16–26°C. Three representative samples of whole plant and rhizosphere soil were collected at 0 (2 h after application), 1, 3, 5, 7, 10, 14, and 21 days intervals after pesticides application. Pepper and eggplant fruit samples were collected on 0, 3, 7, 14, and 21 days.

### Extraction and purification

All samples (plants and fruits) were homogenized using a Philips blender (Shanghai, China) and the ground samples were stored in a freezer (at −20°C) until analysis.

#### Plant samples

The extraction of chlorpyrifos residues from plants and fruits was carried out by the procedure as follows. Five (5.0) g of the homogenized sample was weighed into a 50 mL Teflon centrifuge tube. The extraction solvent (acetonitrile, 10 mL) was added. The samples were then mixed thoroughly for 1 min with a vortex mixer, followed by high-speed homogenizing for about 2 min. After addition of 2 g of sodium chloride, the samples were vortexed immediately for 1 min and centrifuged for 5 min at 5000 rpm. An aliquot of 1 mL of the supernatant was transferred into a 10 mL glass test tube, and then evaporated just to dryness under a stream of nitrogen (40°C). The residue was dissolved in 1 mL of hexane and then subjected to Florisil SPE column clean-up. The SPE column was pre-conditioned by rinsing it with 5 mL of hexane. The extraction was added to the SPE column followed by eluting with 10 mL of a mixture containing acetone and n-hexane (9∶1, v/v). The eluate was evaporated to dryness. The residues were redissolved with acetone to 1 mL. The final extract was filtered through a 0.2 µm SCAA-104 membrane followed by GC analysis.

#### Fruit samples

The method of sample extraction was a modification of a reference method [38]. Fruit sample (5.0 g) was weighted into a polypropylene centrifuge tube. An extraction solvent (10 mL of acetonitrile) was added. The sample was homogenized for 1 min using the homogenizer. The homogenizer probe was rinsed with a portion of 5 mL of the extraction solvent. The extracts were combined. The sample was centrifuged at 5000 rpm for 5 min. Transferred 2 mL of supernatant into a 15 mL centrifuge tube containing PSA (100 mg), ODS-C18 (100 mg) and florisil (100 mg). The sample was vortexed for 1 min and centrifuged at 5000 rpm for 2 min. After centrifugation, the supernatant was filtered using a 0.22 µm nylon filter into an autosampler vial for LC/MS/MS analysis.

#### Soil

Five (5.0) g of soil was weighed into a 50 mL Teflon centrifuge tube and 5 g of sodium chloride was added. The contents were thoroughly mixed. Then, 20 mL of acetonitrile was added. The mixture was vortexed for 1 min, ultrasonically extracted at 40–45°C for 30 min, shaken on a rotary shaker for 2 h, and centrifuged at 5000 rpm for 5 min. Then 1 mL of the supernatant was transferred into a centrifuge tube and dried under a stream of nitrogen (40°C). The chlorpyrifos residues were redissolved in 1 mL of acetone followed by GC analysis.

### GC and LC/MS/MS instrument analyses

#### GC

The conditions for the analysis were: detector temperature, 280°C; injector temperature 270°C; oven temperature program starting at 120°C, 3.67 min at 120–230°C (ramp 30°C min^−1^), 5 min at 230°C, 2 min at 230–270°C (ramp 20°C min^−1^), 2 min at 270°C; carrier gas, N_2_ at 1 mL/min; injection volume 1.0 µL, in a splitless mode. A linear calibration curve was used and the calibration range was 0.01–5 mg kg^−1^. Under these conditions chlorpyrifos retention times were approximately 7.52 min. The software was Agilent ChemStation Rev. B04.03 software for instrument control, data acquisition and processing.

#### LC/MS/MS

The instrument analysis method was an adaption of reference methods [38]. The HPLC conditions for the analysis were: mobile phase A: water containing 0.1% of formic acid (v/v); mobile phase B: acetonitrile containing 0.1% of formic acid (v/v); flow rate 0.4 mL min^−1^; injection volume 10 µL; mobile phase gradients of binary pump: 10% B (0–1.50 min), 95% B (1.51–4.50 min), 95% B (4.51–6.00 min), and 10% B (6.01–7.00 min). The MS/MS conditions were: gas temperature 350°C; gas flow 10 L/min; nebulizer pressure 45 psi; and capillary voltage 4000 V. In order to achieve the highest sensitivity, the fragment, voltage and the collision energy were optimized. MRM (chlorpyrifos) transitions were 350.1>198 (quantitation, collision energy, CE, 20 V) and 350.1>97 (identification, CE 30 V). The retention time was 3.89 min. The software was Agilent MassHunter software for instrument control, data acquisition and processing.

### Data analysis

The degradation rate constant and half-life were calculated using a first-order rate equation:
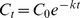
where *C*_t_ and *C*_0_ represent the concentrations of the chlorpyrifos residues at the day *t* and day 0 (2 h), respectively, and k is the degradation rate constant. The half-life (*t*_1/2_) is defined as the time required for the pesticide residue level to fall to the half of the initial residue level of day 0 (i.e., *C*_0_) and was calculated using the following equation:



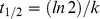



## Results and Discussion

### Method validation

#### GC method

Quantification was accomplished by using the standard curve constructed by plotting analyte concentrations against peak areas. Good linearity was achieved with the chlorpyrifos concentration and the correlation coefficient was 0.9995. Recoveries of chlorpyrifos at different fortification levels, i.e., 0.05, 1, and 10 mg kg^−1^, were determined in three replicates for validation of the method. The recoveries of chlorpyrifos in the soil were 72.5%–89.6% with the relative standard deviation (RSD) 2.1%–7.2% and R^2^ = 0.9846. For the six vegetable plants, the recoveries were from 79.3%–97.0% with RSD 3.0%–15% and R^2^ = 0.9910. The limit of quantification (LOQ) was defined as the lowest fortification concentration whose signal-to-noise (S/N) ratio was equal to or greater than 10 and thus the LOQ of the GC analysis was 0.05 mg kg^−1^. The sample extracts in which the chlorpyrifos residues were greater than 10 mg kg^−1^ were diluted 10 times prior to the evaporation/cleanup steps. And the diluted samples were re-analyzed to ensure the residues were in the acceptable ranges.

#### LC/MS/MS method

Three fortification levels, i.e., 0.01, 0.1, and 2 mg kg^−1^, were analyzed. For pepper fruits, the average recoveries of chlorpyrifos residue in the soil were 87.1%–106% with the relative standard deviation (RSD) from 1.1% to 7.4% and R^2^ = 0.9991. For eggplant fruits, the average recoveries were from 86.1% to 97.0%, with RSD 2.9%–5.9% and R2 = 0.9978. The LOQ of the LC/MS/MS analysis was set to be 0.01 mg kg^−1^.

Soil and whole plant samples of the six selected crops were analyzed using GC (LOQ = 0.05 mg kg^−1^). The fruit samples of pepper and eggplant were analyzed by LC/MS/MS (LOQ = 0.01 mg kg^−1^). These methods were capable of conducting the analyses in this study.

### Degradation of chlorpyrifos

#### Soil

**Figure 1:**
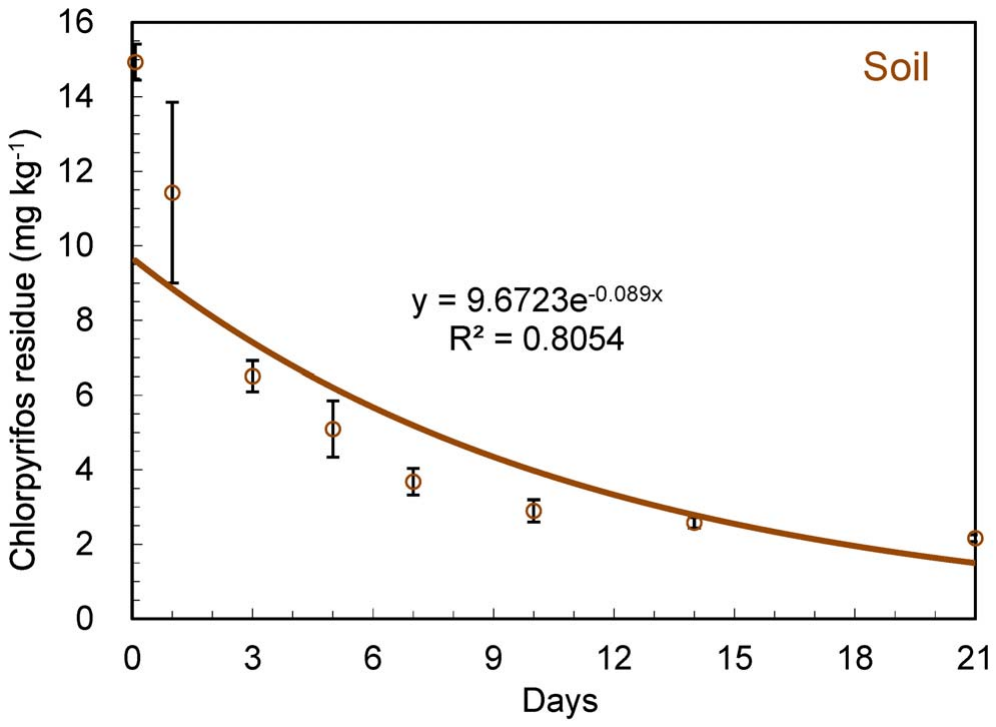
Dissipation dynamic of chlorpyrifos in soil.

The rhizosphere soil samples were collected at different intervals after chlorpyrifos was applied. The chlorpyrifos residues in soil were analyzed by GC. The concentration of chlorpyrifos in the soil decreased over time (Figure 1). The average initial deposition of chlopyrifos was 14.9±0.5 mg kg^−1^ (i.e., 2 h, day 0) and the final residue was 2.2±0.1 mg kg^−1^ on day 21. The first-order kinetic equation of chlorpyrifos dissipation is *C*_t_ = 4.84e^−0.089*t*^ (Table 1) with correlation coefficient R^2^ = 0.8054 and the half-life *t*_1/2_ = 7.79 days. The half-lives of chlorpyrifos in soil were in the range of 3–7 days reported by Singh et al. [30]. Singh et al. observed that chlorpyrifos persisted in a low pH soil, i.e., less than 3% of the pesticide had degraded after 10 days and more than 50% of chlorpyrifos was dissipated at a higher pH soil (pH 8.5). Chai et al. reported that the half-lives in humid tropical soils from Malaysia were typically 7–120 days [32]. However, Chai, et. al. also reported that some half-lives were 257 days in the soils containing less soil microbial populations [32]. In the literature, it was reported long environmental dissipation half-lives of chlorpyrifos, i.e., up to 4 years, depending on application rate, ecosystem, and pertinent environments [33]. Since chlorpyrifos presented low water solubility and a higher log K_ow_, it had a strong tendency to sorb to organic matter and soil. Stability and effectiveness had made chlorpyrifos one of the most popular pesticides worldwide but on the other side its persistence had raised environmental concerns [34].

**Table 1:** Dynamic equations, correlation coefficients and half-lives of chlorpyrifos in soil and six vegetable plants (foliar).

Matrix	Dynamic equation	Correlation coefficient (R^2^)	Half-life (days)
Soil	*C*_t = _9.672 e^−0.089*t*^	0.8054	7.79
Brassica chinensis	*C*_t = _11.74 e^−0.1192*t*^	0.9618	5.81
Lettuce	*C*_t = _17.30 e^−0.1769*t*^	0.9359	3.92
Celery	*C*_t = _40.62 e^−0.1271*t*^	0.9648	5.45
Asparagus lettuce	*C*_t = _31.97 e^−0.1775*t*^	0.8850	3.90
Pepper	*C*_t = _40.23 e^−0.2622*t*^	0.9315	2.64
Eggplant	*C*_t = _48.28 e^−0.2307*t*^	0.9756	3.00

#### Initial Deposition

**Figure 2:**
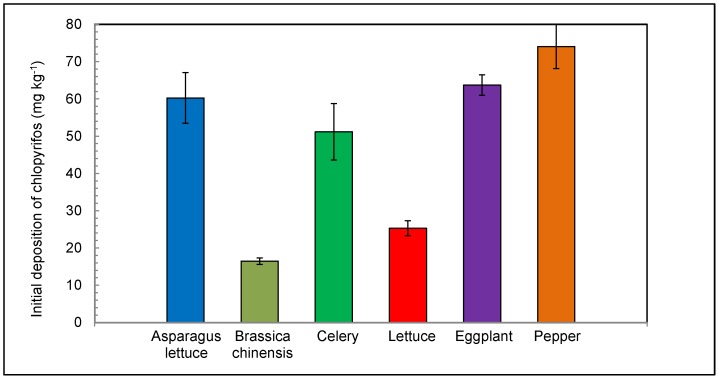
Initial depositions of chlorpyrifos on the six vegetable plants.

After foliar application, the whole plants of the six selected crops were collected. The chlorpyrifos residues in plants were analyzed by GC. The initial depositions of chlorpyrifos in the six selected plants are compared in Figure 2. As can be seen in Figure 2, the initial depositions (2 h, day 0) on the six plants were in an increasing order: 16.5±0.87 mg kg^−1^ (brassica chinensis), 25.3±2.0 mg kg^−1^ (lettuce), 51.2±7.6 mg kg^−1^ (celery), 60.3±6.8 mg kg^−1^ (asparagus lettuce) <63.7±2.8 mg kg^−1^ (eggplant) <74.0±5.9 mg kg^−1^ (pepper), respectively.

It is presumed that the initially deposited chlorpyrifos amount mainly depended upon the surface area of the foliar which the pesticide was sprayed on in spite of the leaf characteristics of the plants, such as leaf roughness, content of cuticular waxes, etc. which were assumed to contribute little to the initial depositions. Therefore, the concentration of the initial deposition is directly proportional to the foliar area and inversely proportional to the biomass of the whole plant. The foliages of pepper plant, eggplant plant, celery, and asparagus lettuce were overlapping to maximize the effective foliar surface area. The shorter plants (lettuce and brassica chinensis) had an area of uncovered soils between rows. As a result, lettuce and brassica chinensis had the lowest initial depositions. Since it was lightest in weight, the pepper plant had over all the largest initial deposition of chlorpyrifos.

#### Dissipation

**Figure 3:**
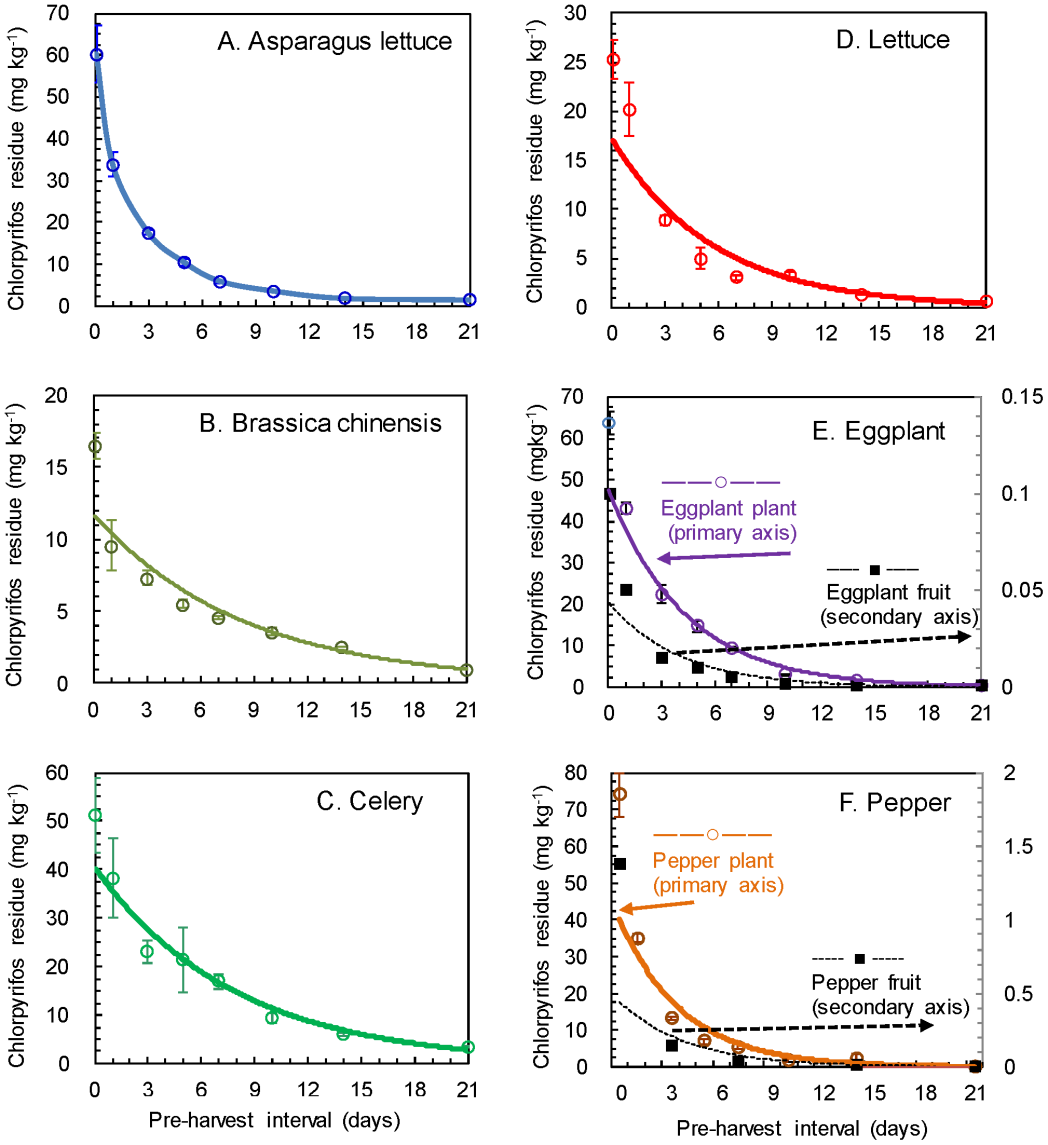
Dissipation dynamic of chlorpyrifos in vegetables and fruits. A. Asparagus lettuce, B. Brassica chinensis, C. Celery, D. Lettuce, E. Eggplant (eggplant plant - solid curve with marker ○ and primary axis; eggplant fruit – dashed curve with marker ▪ and secondary axis), and F. Pepper (pepper plant - solid curve with marker ○ and primary axis; pepper fruit – dashed curve with marker ▪ and secondary axis).

The dynamic equations of chlorpyrifos degradation are given in Table 1. The curves of dissipation in the six plants and two fruits are described in Figure 3. As can be seen in Figure 3, the dynamics curves demonstrated that the chlorpyrifos residues dissipated significantly in the first a few days and persisted in the crops for extended period of time. For example, at pre-harvest interval (PHI) 21 days, the chlorpyrifos residues in the six plants decreased to 1.47±0.22 mg kg^−1^ ((A) asparagus lettuce), 0.97±0.03 mg kg^−1^ ((B) brassica chinensis), 3.50±0.27 mg kg^−1^ ((C) celery), 0.56±0.06 mg kg^−1^ ((D) lettuce), 0.53±0.06 mg kg^−1^ ((E) eggplant), and 0.15±0.01 mg kg^−1^ ((F) pepper), respectively. As can be seen in Table 1, the half-lives (from low to high) were found to be: 0.91 days (pepper plants) <3.92 days (lettuce) <3.92 days (asparagus lettuce) <5.82 days (brassica chinensis) <3.00 days (eggplant plants) <5.46 days (celery).

The possible mechanisms are thought to be due to the difference in the activities of pesticide degradation enzymes and/or pesticide degradation endophytes among these plants [25]. It is interesting to observe that the half-lives of the crop plants seemed to be a reverse order of the initial deposition. Pepper plants had the highest initial deposition but shortest half-live (0.92 day) while brassica chinensis had the lowest initial deposition but longest half-live (5.82 days). The difference in the calculated half-life values indicated that different vegetables had different degradation rates. One of the key factors could be photodegradation of chlorpyrifos [37]. The pepper plant had a greater effective foliar area which led to the highest pesticide position, and exposed to the ultraviolet lights. Also, after application, the leaf characteristics may affect how the pesticide would be retained on the surfaces of the leaves and then be penetrating into the plant tissues such as leaf surface roughness [39]–[40] and the content of water repellent cuticular waxes [41]–[44].

#### Edible parts of the vegetables

The edible parts are edible leaves and stems of brassica chinensis, lettuce, celery, and asparagus, and fruits of eggplant and pepper. The residue in pepper and eggplant fruits were analyzed by LC/MS/MS. The comparison of the residue data are given in Table 2. A number of existing Maximum Residue Limits (MRLs) and crop grouping are also included in Table 2. The higher residues of chlorpyrifos in the plants were due to high initial depositions. It was observed that the residues of foliages were significantly higher than those in the edible fruits (eggplant and pepper fruits), i.e., pepper had 0.03 mg kg^−1^ and <0.01 mg kg^−1^ of chlorpyrifos residues and eggplant had <0.10 mg kg^−1^ at PHI 7 days and PHI 21 days, respectively (Table 2). In Figure 3 (E and F), The half-lives were calculated to be 2.84 days (pepper fruits) and 3.15 days (eggplant fruits), respectively. It was observed that same residues exceeded the MRLs. However, it should be noted that the purpose of this work was to study the residues of chlorpyrifos change in the crops. Therefore, higher application rates were used in order to monitor such changes.

**Table 2:** Comparison of chlorpyrifos residues in edible parts of brassica chinensis, lettuce, celery, asparagus lettuce, eggplant and pepper with Maximum Residue Limits (MRLs).*^1.^

		Residue (mg kg^−1^)		MRL (mg kg^−1^)			
Commodity	Crop group*^2^	PHI 7 days	PHI 21 days	CAC*^3^	China*^4^	USA*^5^	EU*^6^
**Edible plants:**							
Brassica chinensis	5B	4.55	0.97	1.0	0.1	1.0	0.5
Lettuce	4A	3.07	0.56	*^7^	0.1	*^7^	0.05
Celery	4B	16.9	3.50	*^7^	0.05	*^7^	0.05
Asparagus Lettuce	4B	5.96	1.47	*^7^	0.1	*^7^	*^7^
**Edible fruits:**							
Eggplant (fruit)	8–10B 8–10C	0.01	<0.01	*^7^	*^7^	*^7^	0.5
Pepper (fruit)	8–10B	0.03	<0.01	2.0(sweet pepper)	*^7^	1.0	1.0

^*1^Please note that this work intended to study pesticide persistence instead of for MRL establishment. The comparison described in above the table is used to study the persistence and dissipation of chlorpyrifos in the selected crops. The high residues above were due to the high application rate and different formulations.

^*2^Code of Federal Regulations Title 40 Part 180.41 Crop group table (40 CFR 180.41). Group 4A/4B Leafy vegetables (except brassica vegetables), 5B Brassica leafy vegetable, 8–10B 8–10C Fruiting vegetable group.

^*3^CODEX Alimentarius: List of standards: http://www.codexalimentarius.org/standards/list-of-standards/en/?provide=standards&orderField=fullReference&sort=asc&num1=CAC/MRL.

^*4^Chinese National Standards (GB2763-2012): Maximum Residue Limits for Pesticides in Foods.

^*5^The United States Tolerances and Exemptions for Pesticide Chemical Residues in Food: http://www.ecfr.gov/cgi-bin/text-idx?SID=e33dfa87fab3f5ddecad25dafa7028ea&node=40∶25.0.1.1.27.3.19.113&rgn = div8.

^*6^Pesticide EU-MRLs (Regulation (EC) No 396/2005, MRLs updated on 28/01/2014) http://ec.europa.eu/sanco_pesticides/public/index.cfm?event=substance.resultat&s=1.

^*7^MRLs not currently established or registrations canceled.

Because of chlorpyrifos’ high hydrophobicity (high K_ow_ value), the pesticide would readily enter into the inter parts from the surfaces resulting in high residue levels. A portion of the residues may be transferred from leaves to the growing fruits. For eggplant and pepper fruits, the difference in chlorpyrifos residues mainly depended upon the composition of the surface waxes from pepper and eggplant [41], [44]. Bauer et. al. [44] reported that the bell pepper contained 39% of fraction 1 (mainly C20–C35 of alkanes and aldehydes) and 61% of fraction 2 (15 various triterpenes) while the eggplant cultivars had 77% of fraction 1 and 23% of fraction 2. Fraction 2 consisted of 15 triterpenes, including α- and β-amyrin, lupeol, glutinol, 3β-friedelanol, friedelin, taraxerol, taraxasterol, δ-amyrin, germanicol, multi-florenol, ω-taraxasterol, isomultiflorenol, isobauerenol and bauerenol, as well as n-alkanoic acids 2-hydroxy-alkanoic acids [44]. These chemicals would enhance the dissipation of chlorpyrifos.

The comparisons of the chlorpyrifos residue data listed in Table 2 and presented in Figure 3 indicated that chlorpyrifos was relatively stable and persisted in the crops, especially leafy crops. The rate of degradation of pesticide residue is affected by environmental conditions, nature of the pesticide, application rate, formulation, and plant species, etc. [45]. The vegetables selected in this study are minor crops representing a range of various species and it was intended to promote the process. For the crop grouping, celery may be a good reprehensive species of leafy vegetable (group 4B, Table 2) with consideration of initial deposition and dynamic data.

## Conclusions and Implications

The study investigated the residue behavior of chlorpyrifos in six vegetables in the greenhouse. The results of chlorpyrifos of initial depositions on different vegetables detected after pesticide application showed differences among the six selected crops. The half-lives of chlorpyrifos in the six vegetables were different indicating that different vegetables had different capacities for metabolizing chlorpyrifos.
